# The INIA19 Template and NeuroMaps Atlas for Primate Brain Image Parcellation and Spatial Normalization

**DOI:** 10.3389/fninf.2012.00027

**Published:** 2012-12-06

**Authors:** Torsten Rohlfing, Christopher D. Kroenke, Edith V. Sullivan, Mark F. Dubach, Douglas M. Bowden, Kathleen A. Grant, Adolf Pfefferbaum

**Affiliations:** ^1^Neuroscience Program, SRI InternationalMenlo Park, CA, USA; ^2^Oregon National Primate Research Center, Oregon Health & Science UniversityBeaverton, OR, USA; ^3^Department of Psychiatry and Behavioral Sciences, Stanford UniversityStanford, CA, USA; ^4^National Primate Research Center and Department of Psychiatry and Behavioral Sciences, University of Washington School of MedicineSeattle, WA, USA

**Keywords:** brain atlas, minimum-deformation template, rhesus macaque (*Macaca mulatta*), magnetic resonance imaging, NeuroMaps

## Abstract

The INIA19 is a new, high-quality template for imaging-based studies of non-human primate brains, created from high-resolution, *T*_1_-weighted magnetic resonance (MR) images of 19 rhesus macaque (*Macaca mulatta*) animals. Combined with the comprehensive cortical and sub-cortical label map of the NeuroMaps atlas, the INIA19 is equally suitable for studies requiring both spatial normalization and atlas label propagation. Population-averaged template images are provided for both the brain and the whole head, to allow alignment of the atlas with both skull-stripped and unstripped data, and thus to facilitate its use for skull stripping of new images. This article describes the construction of the template using freely available software tools, as well as the template itself, which is being made available to the scientific community (http://nitrc.org/projects/inia19/).

## 1 Introduction

An image-based atlas typically provides one or several template images displaying the anatomy of interest using one or several imaging modalities, such as microscopy or magnetic resonance imaging (MRI). These template images can be used for aligning the atlas with new images for the purpose of normalizing observed data into common coordinate system (Ashburner and Friston, 2000). In addition, regions of interest can be outlined in the template images and propagated from these to the observed data (Gee et al., 1993; Fedorov et al., 2011).

A number of MRI-based non-human primate brain atlases are available, including the baboon (Black et al., 2001b) and macaque MRI/PET templates (Black et al., 2001a), the rhesus macaque atlas (McLaren et al., 2009), the common marmoset atlas (Hikishima et al., 2011), the MNI macaque template (“MNI Monkey Space”; Collantes et al., 2009), and the NeuroMaps macaque atlas (Kuperman et al., 2006; Bowden and Annese, 2007; Bowden et al., 2007; Dubach and Bowden, 2009). Table 1 provides an overview of image-based atlases for non-human primate brains that are currently available for download, in roughly chronological order.

**Table 1:** **Overview of publicly available MRI-based non-human primate brain atlases**.

Atlas name	Animals	Reference	Channels	Template resolution	Normalization	Compared with INIA19
N2K	*Macaca nemestrina* (*n* = 12)	Black et al. (2001b)^1^	*T*_1_ MRI, ${\text{H}}_{2}^{15}\text{O PET}$	1.0 × 1.0 × 1.0 mm	Affine	No non-rigid normalization; no tissue segmentation; no parcellation maps
B2K	*Papio* spp. (*n* = 9)	Black et al. (2001b)^2^	*T*_1_ MRI, ${\text{H}}_{2}^{15}\text{O PET}$	1.0 × 1.0 × 1.0 mm	Affine	No non-rigid normalization; no tissue segmentation; no parcellation maps
NeuroMaps macaque atlas	*Macaca mulatta* (*n* = 1)	Dubach and Bowden (2009)^3^	*T*_2_ MRI, 502-region parcellation map	0.15 × 0.15 × 0.15 mm	Non-linear midline flattening; reflection of one hemisphere to produce symmetric hemispheres	No minimum-deformation template
112RM-SL	*Macaca mulatta* (*n* = 112)	McLaren et al. (2009)^4^	*T*_1_ and *T*_2_ MRI, tissue probabilities	0.5 × 0.5 × 0.5 mm	Affine	No non-rigid normalization; no parcellation maps
None	*Chlorocebus pygerythrus* (*n* = 10)	None^5^	*T*_1_ MRI, tissue probabilities, selected sub-cortical ROI probabilities	0.47 × 0.47 × 0.5 mm	Non-rigid	Only minimal sub-cortical parcellation
None	*Macaca fascicularis* (*n* = 24)	Collantes et al. (2009)^6^	MRI, ^11^C-DTBZ and ^18^F-DOPA PET	1.0 × 1.0 × 1.0 mm	Affine	No non-rigid normalization; no tissue segmentation; no parcellation maps
Japanese macaque atlas	*Macaca fuscata* (*n* = 16)	Quallo et al. (2010)^7^	*T*_1_-weighted MRI, tissue probabilities	0.5 × 0.5 × 0.5 mm	Non-rigid	Single-animal *T*_1_-weighted template; no parcellation maps
none	*Callithrix jacchus* (*n* = 22)	Hikishima et al. (2011)^8^	*T*_1_ MRI, tissue probabilities	0.25 × 0.25 × 0.25 mm	Non-rigid	No parcellation maps
MNI macaque atlas	*Macaca fascicularis* (*n* = 18)	Frey et al. (2011)^9^	*T*_1_ MRI, Paxinos labels	0.25 × 0.25 × 0.25 mm	Non-rigid	No brain-only template; no tissue segmentation; single-hemisphere gray matter parcellation only
	*Macaca mulatta* (*n* = 7)					
INIA19	*Macaca mulatta* (*n* = 19)	This article^10^	*T*_1_ MRI, tissue probabilities, bilateral 724-region NeuroMaps parcellation map	0.5 × 0.5 × 0.5 mm	Non-rigid	

Download links for the atlas data:

^1^http://www.nil.wustl.edu/labs/kevin/ni/n2k/

^2^http://www.nil.wustl.edu/labs/kevin/ni/b2k/

^3^http://braininfo.rprc.washington.edu/TemplateNeuroMaps.aspx

^4^http://brainmap.wisc.edu/monkey.html

^5^http://www.bsl.ece.vt.edu/index.php?page=vervet-atlas

^6^http://www.cima.es/labs-en/instrumental-techniques-micropet/technologies/1

^7^http://brainatlas.brain.riken.jp/jm/modules/xoonips/listitem.php?index_id=9

^8^http://brainatlas.brain.riken.jp/marmoset/

^9^http://www.bic.mni.mcgill.ca/ServicesAtlases/Macaque

^10^http://nitrc.org/projects/inia19/

The rhesus macaque (*Macaca mulatta*, an old-world non-human primate species) is the most common non-human primate species used in neuroscience research. Differences in the brain between the rhesus and other *Macaca* species are related primarily to size. Using an *M. fascicularis* template and a standard set of intracerebral landmarks (Bowden and Dubach, 2000) quantified the distortion necessary to align equivalent landmarks of brains from different species and found that the brains of several other species (including *M. mulatta*, *M. fuscata*, and *Papio*), when adjusted for size, were sufficiently similar to justify using the same template. On the other hand, evidence from human imaging studies suggests that the accuracy of volumetric image registration benefits significantly from using spatial normalization to templates that closely match the average of the population under study (Shen et al., 2007; Huang et al., 2010). Such findings argue in favor of using templates from an atlas approximating the average topology of a given species to achieve optimal normalization accuracy in volumetric image registration.

Our own imaging-based studies rely on automatic three-dimensional image-to-image registration, typically between MR images, rather than landmark-based semiautomatic mapping, as is common when mapping data from two-dimensional histological slides. Therefore, we required an atlas that provided all of the following:

(1) *Structural minimum-deformation template image created via non-rigid spatial normalization*: minimum-deformation templates, on average, tend to minimize the registration error when normalizing new images to the template. The use of non-rigid alignment in template creation also leads to more well-defined anatomy than linear, affine alignment, and the resulting templates are more suitable for use with state-of-the-art non-rigid subject-to-atlas alignment techniques (e.g., Ashburner, 2007; Avants et al., 2008) to achieve optimal alignment quality (Yeo et al., 2008).
(2) *Tissue probability maps*: for each pixel, these represent the probability of presence of either gray matter (GM), white matter (WM), or cerebrospinal fluid (CSF). When mapped onto new images, these maps can be used as segmentation priors to improve tissue classification, particularly in the presence of image noise (Zhang et al., 2001).
(3) *Detailed cortical and sub-cortical parcellation map*: these maps are useful for region-based analyses, both in template space and native space of new images, for example to quantify relative tissue volume per anatomical region (e.g., Sullivan et al., 2011).
(4) *Whole-head template in addition to brain-only template*: as robust, automated skull stripping methods for non-human primate images are not available, having a whole-head template image allows for the co-registration with unstripped images, and can also be used for stripping by propagating the template brain mask onto new images.

None of the existing non-human primate brain atlases satisfied all these requirements (rightmost column in Table; Ashburner, 2007), thus motivating the creation of a new template and cortical atlas for imaging-based studies, specifically studies of *M. mulatta*.

The new template, named INIA19 after the Integrative Neuroscience Initiative on Alcoholism^1^ and for its use of images from 19 animals, was created using state-of-the-art techniques for construction of unbiased template images by reference-free non-rigid image registration. The newly created template was then combined with the existing NeuroMaps label map (Kuperman et al., 2006; Bowden et al., 2007) to provide a powerful tool for studies requiring both spatial normalization and label propagation.

Herein, we describe the methods and data employed to construct the INIA19 *T*_1_-weighted brain and whole-head template images, tissue probability maps, as well as the comprehensive cortical and sub-cortical label map. All software tools used for atlas construction are freely available, and the INIA19 atlas is being made available to the scientific community.

## 2 Materials and Methods

Below, we describe the methods and data used to construct the INIA19 template images and its associated NeuroMaps label map. Image analysis software used for creation of the INIA19 atlas included the Computational Morphometry Toolkit (CMTK) for DICOM stacking, intensity bias correction, image alignment, groupwise registration, reformatting, and general-purpose image operations (e.g., averaging). CMTK is available from http://nitrc.org/projects/cmtk/. In addition, we used FSL’s Brain Extraction Tool (BET; Smith, 2002) for brain mask creation and the FAST tool (Zhang et al., 2001) for tissue segmentation. FSL is available from http://www.fmrib.ox.ac.uk/fsl/.

### 2.1 Animals

The animals used to construct this atlas were 20^2^ young to middle-aged male adults, chosen to have no common parents or grandparents, from the pedigreed Oregon National Primate Research Center breeding colony. The mean ± SD in age was 8.0 ± 1.6 years, distributed over a range from 6.2 to 10.5 years. For approximately 6 months prior to imaging, each monkey was individually housed in a stainless steel cage measuring 1.6 × 0.8 × 0.8 m (Allentown Caging, Allentown, NJ, USA) in a vivarium with a 12 h light/dark cycle (with lights on at 7 a.m.) that was maintained at 21 ± 1°C and 30–50% humidity. Each monkey had visual, auditory, and olfactory access to other monkeys in the vivarium, and limited physical access to a neighboring monkey. The monkeys were fed a diet of fresh fruit and 1 g banana-flavored pellets (consisting of 63% carbohydrate, 4% fat, and 22% protein PJ Noyes, Lancaster, NH, USA) in quantities sufficient to maintain a positive caloric intake.

All animal procedures were conducted in accordance with the “Guidelines of the Committee on the Care and Use of Laboratory Animal Resources” (National Health Council, Department of Health, Education, and Welfare, ISBN 0-309-05377-3, revised 1996). Prior to their implementation, procedures were reviewed by the Institutional Animal Care and Use Committee of the Oregon National Primate Research Center and were in compliance with all local, state, and national regulations pertaining to the humane use of animal subjects.

### 2.2 Imaging

Magnetic resonance data were acquired using the same general data acquisition protocol, described in an earlier report (Flory et al., 2010). Following a 12-h fast, the monkeys were removed from their home cages under 10 mg/kg of ketamine anesthesia (Vedco, St. Joseph, MO, USA) and transported to the adjacent MRI facility (under 5 min transport time). At the MRI facility, an endotracheal tube was inserted and anesthesia was maintained by the inhalation of 1.5% isoflurane gas (Butler Animal Health Supply, Dublin, OH, USA) and oxygen (Polar Cryogenics Inc, Portland, OR, USA).

The monkeys were then placed inside a Siemens Trio whole-body 3T MRI system (Erlangen, Germany). For 12 animals, their heads were positioned in the center of a circularly-polarized extremity transmit/receive RF coil. For the remaining 8 animals, a circularly-polarized transmit, 8-element receive (*InVivo*, Orlando, FL, USA) RF coil was used instead.

After positioning the monkey in the scanner, a set of five to six *T*_1_-weighted MP-RAGE images (TE = 4.38 ms, TR = 2500 ms, TI = 1100 ms) were obtained in 7 min 58 s acquisition time per image. For each image, the number of averages (NEX) was equal to 1, voxel sizes were 0.5 mm isotropic, and 128 slices were acquired. In-plane image sampling consisted of 128 and 96 data points in the readout and phase-encode directions, respectively, with no sub-sampling or acceleration factors. The signal-to-noise ratios of images obtained with the two RF coils, averaged over the brain, were within 10% of each other.

Images from all animals were inspected for quality and gross anatomical malformations that would render the images unsuitable for atlas generation. As a result of this survey, one animal was excluded from further processing due to abnormal posterior lateral ventricle anatomy (an image of this animal’s brain with the abnormality visible can, however, be seen in Figure 9 below). A total of 111 images acquired from the remaining 19 animals was thus incorporated into the INIA19 atlas.

### 2.3 Image preprocessing

**Figure 1:**
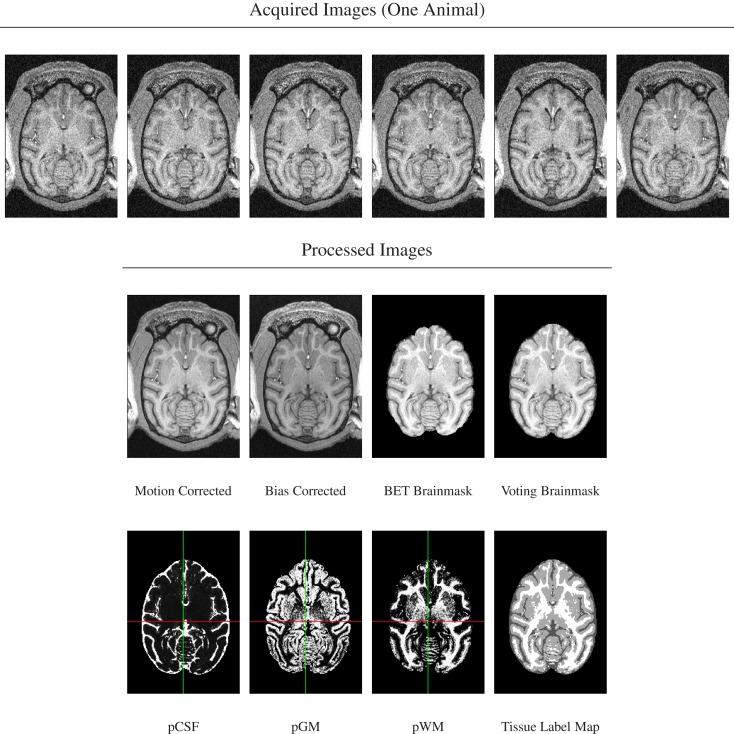
**Image preprocessing stages illustrated for scans from one animal**. *Top row*: six acquired *T*_1_-weighted images. Note presence of motion as evidenced by subtle change of cut plane through eye balls. *Bottom rows*: processed images in order of processing. Note re-added posterior brain tissue when using voting brain mask compared with single-animal BET brain mask.

A series of image processing operations was first applied to all acquired MR data to correct various types of artifacts, extract brain masks, and compute tissue probability maps. These stages of image preprocessing are illustrated for one animal in Figure 1 and each stage is described in more detail below.

#### 2.3.1 Motion correction

For each animal, the first acquired *T*_1_-weighted image was selected as a reference and the remaining images from that animal were aligned to it by rigid (6 degrees of freedom) registration using CMTK’s registrationx tool with three-level multi-resolution optimization of the Mean Squared Difference (MSD) similarity measure and cubic interpolation. The aligned images were then reformatted using the derived rigid-body transformation parameters and cosine-windowed sinc. All images – reference and reformats – were averaged to obtain the motion-corrected images.

#### 2.3.2 Intensity bias correction

Intensity bias was corrected by applying a multiplicative, second-order polynomial bias field to each motion-corrected image. The bias field was computed via entropy minimization (Likar et al., 2001), as implemented in CMTK’s mrbias tool.

#### 2.3.3 Skull stripping

**Figure 2:**
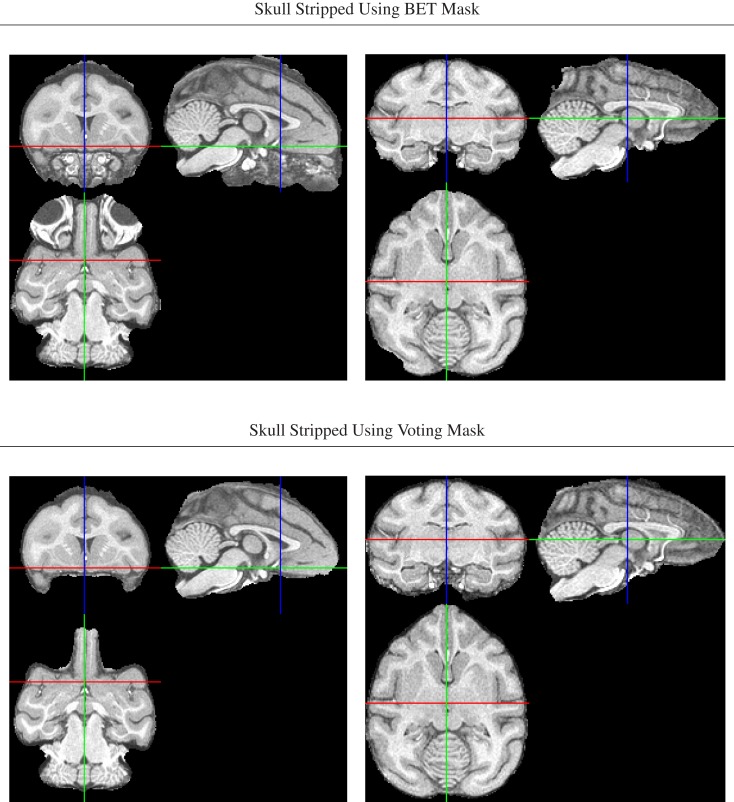
**Illustration of improved brain masks from voting compared with BET**. *Top row*: images from two of the animals for which BET produced sub-optimal masks, one with large non-brain areas remaining and one with brain areas (anterior and posterior) removed. *Bottom row*: both problems are largely fixed using the voting bootstrap procedure described in the text.

An initial brain mask was first generated for each intensity bias-corrected image using FSL’s Brain Extraction Tool, BET (Smith, 2002), but the resulting masks were found to be of insufficient quality due to a combination of both missing brain areas and still unremoved non-brain areas (Figure 2).

To improve brain mask quality, all unstripped images were registered to one another using pairwise image registration as implemented in CMTK’s registrationx (affine) and warpx (non-rigid) registration tools. For each image, the initial brain masks of all other images were then reformatted to it and combined into the final brain mask using label voting, i.e., a pixel was classified as “brain” if the majority of the 19 initial masks (1 animal-native, 18 reformatted) classified it as such, and as background otherwise.

### 2.4 Brain template creation

Creation of the brain image template used the same reference-free unbiased groupwise non-rigid image registration approach (Learned-Miller, 2006; Balci et al., 2007) previously used to create the SRI24 atlas of the human brain (Rohlfing et al., 2010). Specifically, the following three stages of progressive alignment were performed:

(1) Initial translational alignment of all input images based on their centers of mass, using CMTK’s groupwise_init tool.
(2) Affine alignment (rigid plus anisotropic scale factors), using the groupwise_affine tool.
(3) Full non-rigid alignment using multi-level B-spline free-form deformation (Rueckert et al., 1999), using the groupwise_warp tool.

All three stages enforced a zero-sum constraint for each transformation parameter over all 19 individual images. The resulting average coordinate space represents a form of “minimum-deformation template” (Kochunov et al., 2001), i.e., one that requires the least amount of deformation on average to align it with a typical population of images.

### 2.5 Whole-head template creation

Although the brain is clearly the focus of interest for the vast majority of imaging-based neuroscience studies, it is advantageous to have a whole-head template image also available. This facilitates the alignment of individual data sets from which the non-brain tissue has not been removed (i.e., images that have not been skull-stripped). In short, an atlas that provides both brain and whole-head template images can be used equally with and without skull-stripped data.

Creation of a template from unstripped images would be ill-advised, however, because the presence of non-brain tissue can interfere with image registration (Battaglini et al., 2008). Thus, by creating a template from unstripped images only would compromise the quality of the template in the brain areas. At the same time, creating whole-head and brain templates separately and compositing them would produce discontinuities between brain and non-brain regions in the combined whole-head image.

**Figure 3:**
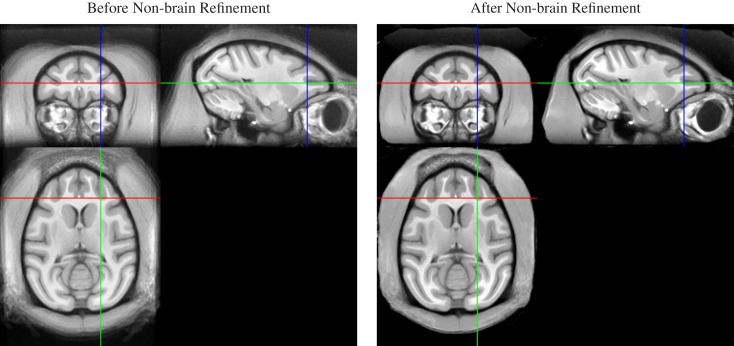
**Whole-head average image before (left) and after (right) local non-brain refinement**. Note that appearance of the brain does not change as a result of constraining the registration to affect only non-brain areas. The result is a whole-head template image that maintains quality of the brain regions achieved by alignment of skull-stripped images, but also provides a consistent embedding of that brain template image into the whole-head template.

Thus, we applied a sequential approach to create both brain and whole-head templates, in this order, by adding a second non-rigid normalization stage after the non-rigid averaging of skull-stripped images. In this second stage, we simultaneously registered all whole-head images of the 19 input animals, but (a) initialized the registration transformation for each animal with the result of the brain-only registration, and (b) fixed the transformation in the brain region. As a result, the previously aligned brains remained in alignment, while the only coarsely aligned (via spatial proximity and smoothness of the registration) non-brain areas were selectively refined (see Figure 3).

### 2.6 Template alignment

To provide anatomically interpretable image coordinates, the final brain template was re-aligned from its original, essentially random position and orientation. To this end, the midsagittal plane was first computed using CMTK’s symplx tool as the plane of maximal symmetry (Citardi et al., 2004). A rigid coordinate transformation was created that aligned this plane with the mid-plane of the template image grid.

One of the authors (Christopher D. Kroenke) then manually identified the locations of the anterior commissure (AC) and posterior commissure (PC) landmarks, each defined as the center of the commissure at the midline, in the brain template image. A rotation transformation was determined that brought both landmarks into the same axial plane and also rotated the PC to a location exactly posterior from the AC. All atlas image files were then re-generated, with the symmetry and AC/PC alignment transformations concatenated onto the front of any other normalization transformations, to obtain final images with only a single interpolation from the original image data.

Finally, the image-to-physical space transformation matrices stored in all NIFTI image files of the different atlas images were adjusted such that the AC landmark became the origin (i.e., zero coordinate) of the image coordinate system. This adjustment should facilitate the use of the INIA19 template images in common neuroimaging software such as SPM (Friston et al., 2007)^3^.

#### 2.6.1 Tissue segmentation

For each animal, its bias-corrected and skull-stripped *T*_1_-weighted image was segmented into three tissue compartments (CSF, cerebrospinal fluid; GM, gray matter; WM, white matter) using FSL’s FAST tool (version 4.1). The tissue probability maps were then reformatted into template space using linear interpolation and averaged. From the three averaged probability maps, the maximum-likelihood (ML) tissue segmentation was then determined by classifying each pixel according to the tissue class with the highest averaged probability over all animals at that pixel (Figure 1, bottom row).

### 2.7 NeuroMaps labeling

The NeuroMaps rhesus macaque brain atlas^4^ consists of an MRI template image and a detailed cortical and sub-cortical label map for that image. Since this template itself has not been fully described in the literature, we first briefly summarize its creation below.

The MRI template is an image of the *ex vivo* brain of a single, 3-year-old male rhesus macaque obtained from the Russian primate center at Sochi, Russia. The brain was first perfused with papaverine and heparin to prevent clotting during perfusion with paraformaldehyde for fixation. The specimen was then immersed in 1 mM gadopentetate dimeglumine, Gd-DTPA (Magnevist), for several days to enhance gray-white contrast. For scanning, the specimen was suspended in cotton batting in a cylindrical canister, which was filled with Fomblin to minimize image noise in the surrounding medium and pressurized to prevent artifacts due to air bubbles. A high-resolution MR image with 0.15 mm^3^ isotropic spatial resolution was acquired on a 4.7T Bruker Biospec Avance scanner with a three-dimensional *T*_2_-weighted gradient echo sequence.

The imaged brain was then made symmetric by flattening the brain’s mid-plane and reflecting one hemisphere with respect to the flattened mid-plane as described by Bowden et al. (2011) for an analogous mouse brain atlas creation procedure.

The cortical and sub-cortical label map was created manually by one of the authors (Mark F. Dubach), outlining structures on coronal image sections and initializing each subsequent slice by copying the regions outlined in the previous one. Discontinuities were then edited in orthogonal cross-sections. A total of 502, pixel-wise mutually exclusive regions were thus labeled in the map used herein.

The NeuroMaps label map, while arguably the most complete and accurate digital segmentation available, is still being actively improved^5^. Currently, confidence in the match of structural boundaries defined on the basis of the MRI to boundaries found in conventional atlases based on Nissl-stained sections varies for different parts of the brain. We have confidence in the current representation of the classical cortical structures (e.g., gyri and lobules), cortical white matter, major myelinated tracts, basal ganglia, ventricles, and cerebellum. The segmentations of the basal forebrain, hypothalamus, and amygdala are incomplete. The segmentations of the midbrain and hindbrain have not been fully reviewed. The incomplete internal segmentation of the thalamus is not considered reliable at this time, but this labeling will be replaced in the near future with boundaries mapped from the atlas by Ilinsky et al. (2002).

**Figure 4:**
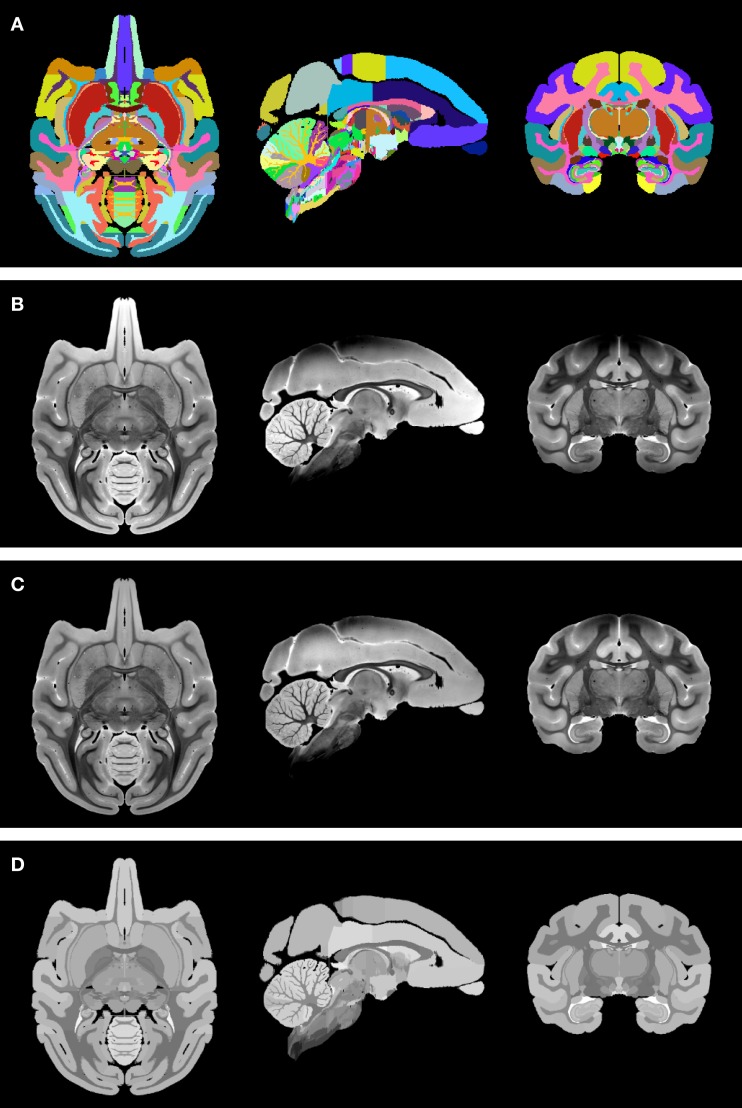
**Preparation of the NeuroMaps label map and MR image: (A) symmetric, whole-brain label map**. The two hemispheres use distinct labels, but left and right copy of each region are shown here using the same color for simplicity. **(B)** Symmetric, whole-brain MR image as provided through the NeuroMaps website. **(C)** Intensity bias-corrected MR image. **(D)** Region-averaged MR image. See text for details.

To transfer the label map to the newly created INIA19 template space, the NeuroMaps data were prepared as follows:

(1) To be able to distinguish left from right hemispheric regions in the label map image, the original NeuroMaps assignment of region names to label values was kept for the brain left hemisphere, whereas 1000 was added to each label value for the right hemisphere (but note that, for simplicity, Figure 4A uses the same colors to visualize the two hemispheres).
(2) The MR image (Figure 4B) was corrected for intensity bias by applying a multiplicative, second-order polynomial bias field computed via entropy minimization (Likar et al., 2001; result shown in Figure 4C).
(3) For further reduction of local shading variations, which could interfere with subsequent image alignment, the MR image intensities were averaged over each of region of identically labeled pixels in the label map separately. All pixels within each region were set to the mean intensity over that region to create a region-averaged MR image (Figure 4D).

The region-averaged NeuroMaps MR image was then used for structural image alignment with the INIA19 MR template image and transfer of the label map to the INIA19 template space. It is now well-established, that the quality of label map propagation is greatly increased when using multiple source atlases (Rohlfing et al., 2004a,b; Rohlfing and Maurer, 2005), a process commonly referred to as multi-atlas segmentation and label fusion. Here, however, we have only one source atlas available, so we chose instead a method suggested by Heckemann et al. (2006) as “indirect fusion” and used by us also in creating the SRI24 atlas of the human brain (Rohlfing et al., 2010).

We used all 19 animals that were used to create the INIA19 template as intermediates, because their images were already aligned to the template space by construction. The image data set of each animal was also co-registered with the region-averaged NeuroMaps MR image via non-rigid registration using CMTK’s warpx tool. The NeuroMaps label map was then propagated 19 times, once per intermediate animal, into INIA19 space and all 19 propagated maps were finally combined using shape-based averaging (Rohlfing and Maurer, 2007) as implemented in CMTK’s sba tool.

Given the lower resolution of our *in vivo* MR images, and thus the INIA19 template image, relative to the NeuroMaps MR image and label map, 362 of the 502 manually outlined unilateral labels survived bilateral projection, mapping to INIA19 space, label map averaging, and down-sampling. As a result, there are 724 unique bilateral labels in the INIA19-mapped NeuroMaps parcellation map, one left/right pair for each of the surviving 362 regions.

## 3 Results

The following is a detailed description of the INIA19 atlas as it is being made available to the scientific community via the atlas project web page at http://nitrc.org/projects/inia19/.

### 3.1 Image file format and coordinates

**Figure 5:**
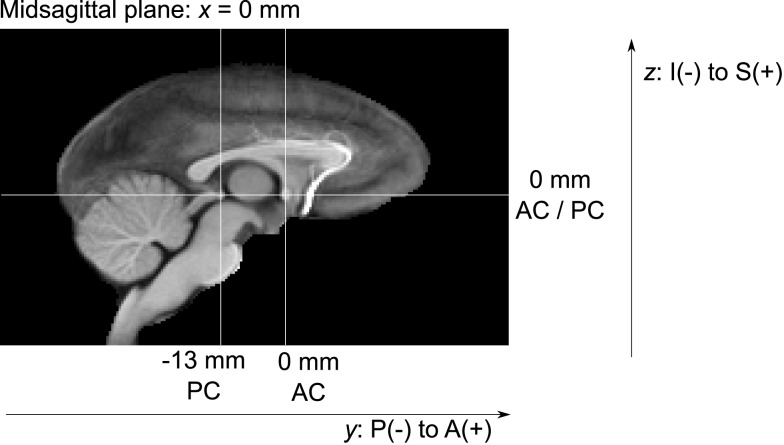
**Coordinate system defined by the AC and PC landmarks in the midsagittal plane (x = 0 mm)**.

All atlas files are distributed in NIFTI-1 data format^6^, stored in “RAS” index order^7^, with a grid size of 168 × 206 × 128 pixels and 0.5 mm isotropic pixel size. The template coordinate space is aligned with the image grid such that the midsagittal plane of the template anatomy coincides with the mid-plane of the image grid. The physical image coordinates, represented as direction vectors and offset in the NIFTI headers, were adjusted such that the zero physical space coordinate coincides with the AC landmark, and the PC landmark is exactly posterior from PC (see Figure 5 for illustration).

The distributed files are:

**inia19-t1.nii:** Whole-head *T*_1_-weighted template image.
**inia19-brainmask.nii:** Binary mask of the brain.
**inia19-t1-brain.nii:** *T*_1_-weighted template image of the brain with non-brain tissue removed.
**inia19-prob_CSF.nii:** CSF tissue probability map.
**inia19-prob_GM.nii:** Gray matter tissue probability map.
**inia19-prob_WM.nii:** White matter tissue probability map.
**inia19-tissue.nii:** Maximum-likelihood tissue label map (0 = background, 1 = CSF, 2 = GM, 3 = WM).
**inia19-NeuroMaps.nii:** Bilateral NeuroMaps parcellation label map (labels 1–605: left hemisphere; labels 1001–1605: right hemisphere). An accompanying text file, inia19-NeuroMaps.txt, provides a list of all label values, region names, and RGB colors as assigned in the NeuroMaps atlas. This file can be used directly with 3D Slicer^8^ for visualization.

### 3.2 Atlas channels

**Figure 6:**
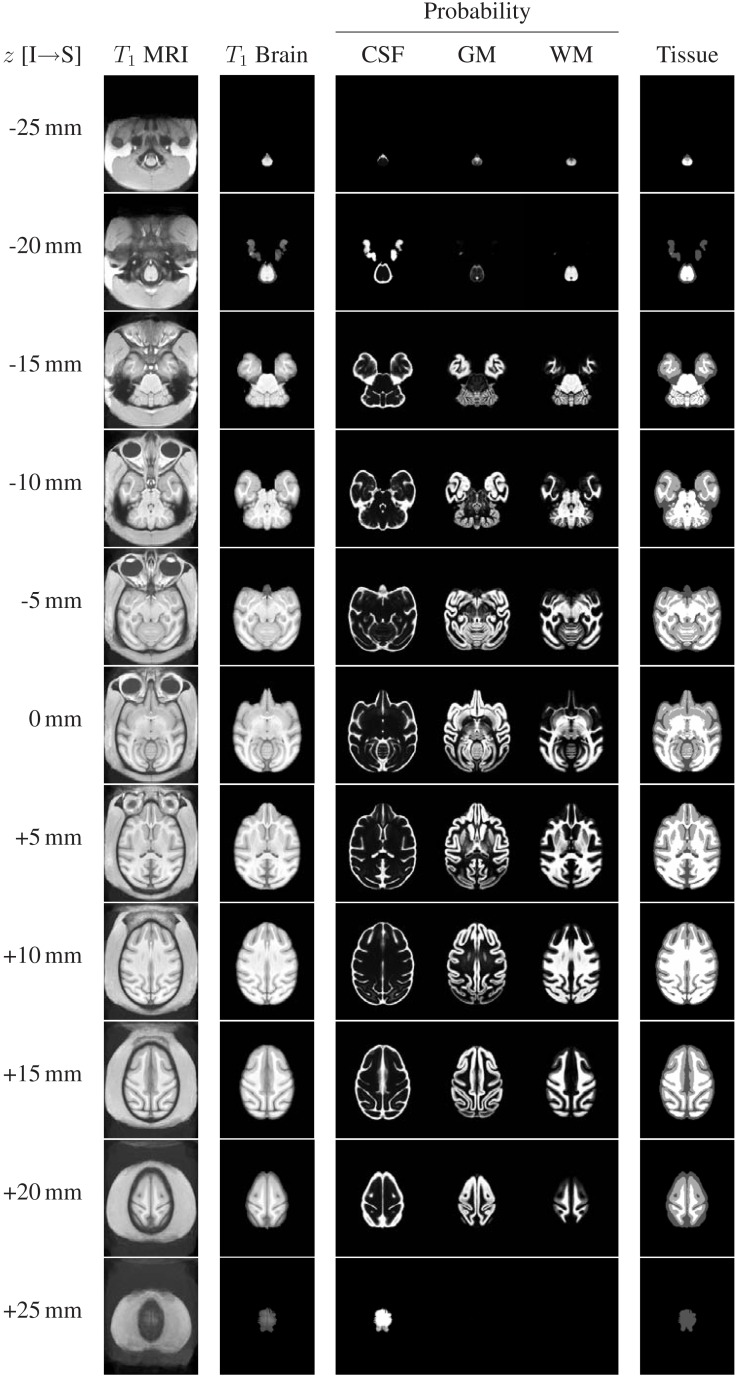
**Representative axial slices from the INIA19 atlas images (every tenth slice is shown)**. The slice location (*z* coordinate in NIFTI RAS coordinate space) is provided in the leftmost column. The AC and PC landmarks are both located in the *z* = 0 mm slice.

The structural channels of the INIA19 atlas (*T*_1_-weighted whole-head and brain-only MRI, tissue probabilities, and ML tissue labels) are shown in Figure 6 for axial slices with 5 mm spacing (recall that the actual template images have 0.5 mm slice spacing).

**Figure 7:**
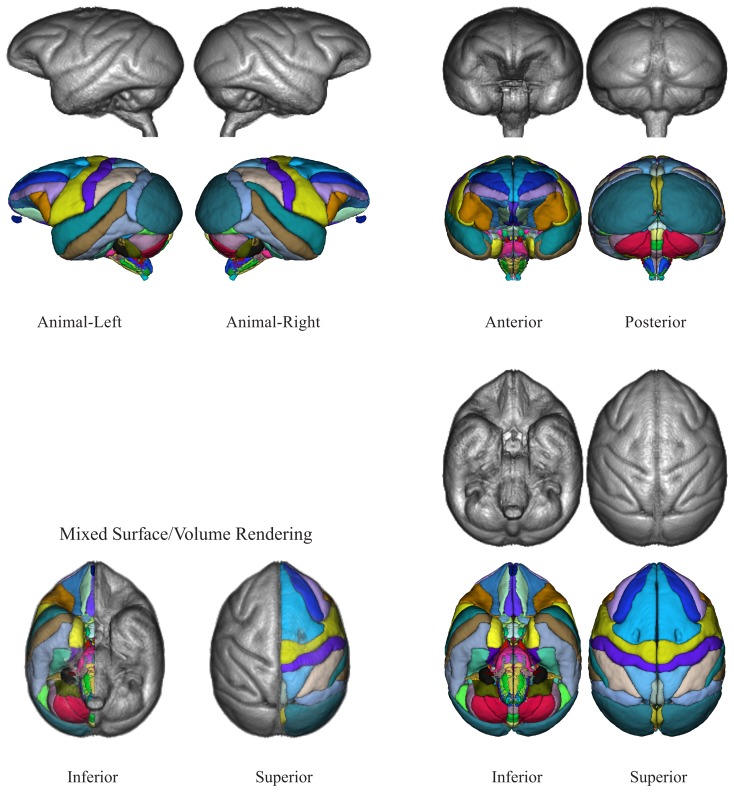
**Three-dimensional volume renderings of the T1-weighted INIA19 brain template image (gray) and surface renderings of the NeuroMaps label map (colors) after mapping into INIA19 coordinate space**.

**Figure 8:**
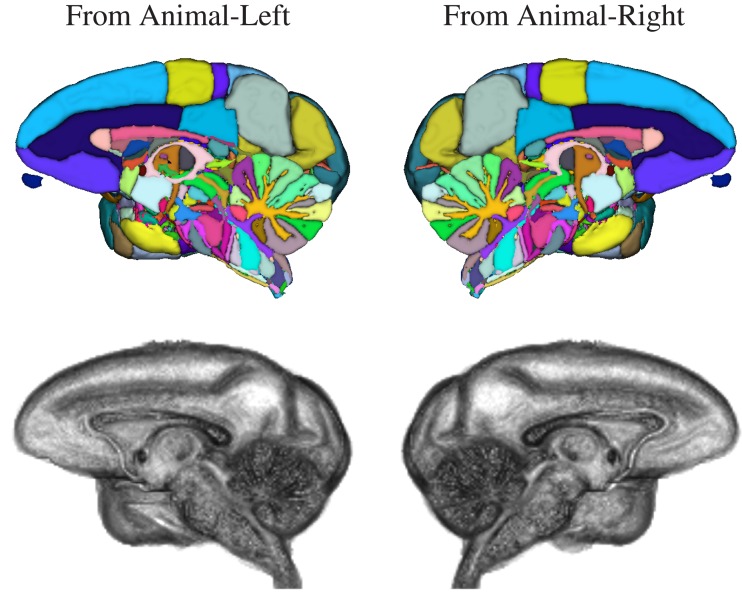
**Three-dimensional volume renderings of the T1-weighted INIA19 brain template image (gray) and surface renderings of the NeuroMaps label map after mapping into INIA19 space (colors), each cut at the midsagittal plane to reveal the proximal surface of the opposite hemisphere**.

The NeuroMaps labeling of the INIA19 brain template image is visualized in Figures 7 and 8. (A label description file containing label index, anatomical structure name, and RGB color information matching these renderings is provided with the atlas.)

## 4 Applications

Next, we demonstrate the use of the INIA19 atlas for the two typical atlas applications, (a) propagation of atlas information to individual animals, and (b) spatial normalization of individual images to the atlas.

### 4.1 Atlas propagation

**Figure 9:**
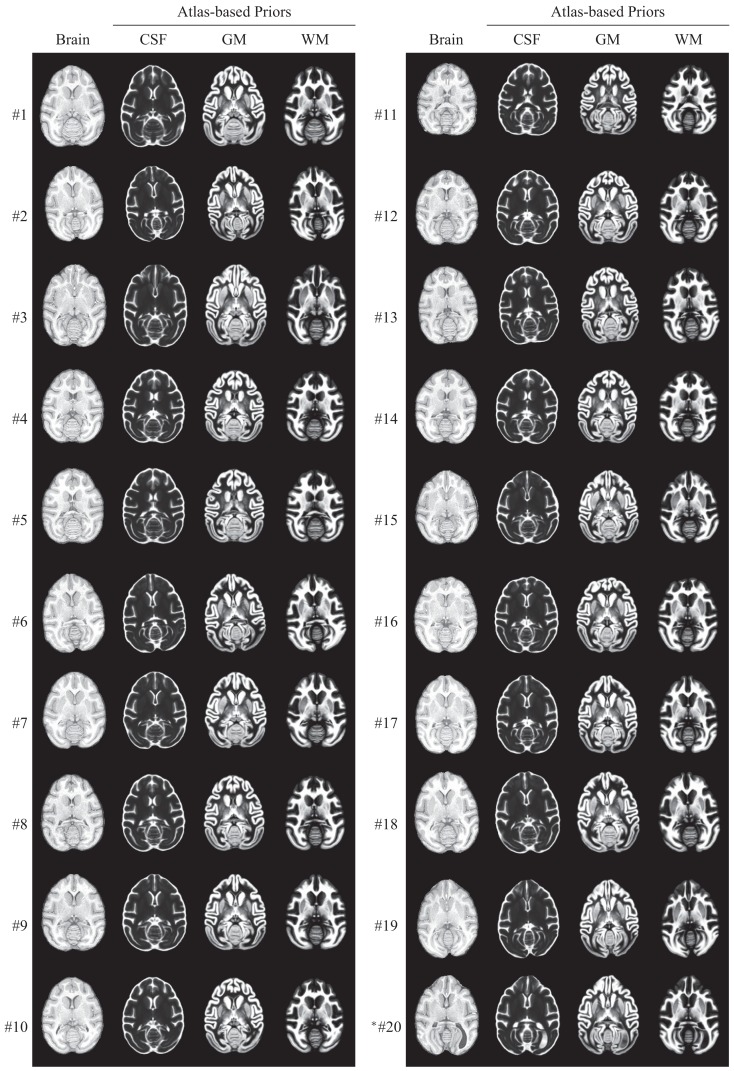
**INIA19 tissue priors reformatted to each of 20 animal images via non-rigid registration**. *Animal #20 was the one that was excluded from atlas generation due to abnormal ventricular anatomy, which is clearly visible in the posterior right (animal-left) hemisphere.

After computing a coordinate transformation, via image registration, from an individual animal image to the atlas template image, data such as labels can be mapped back from the atlas into the individual image. For the INIA19 atlas, this includes specifically tissue labels and probabilities as well as NeuroMaps labels. In Figure 9, we demonstrate specifically the usefulness of the atlas tissue probabilities, mapped to each of our 20 originally imaged animals (including the one excluded from atlas construction), as priors for tissue segmentation.

### 4.2 Spatial normalization

**Figure 10:**
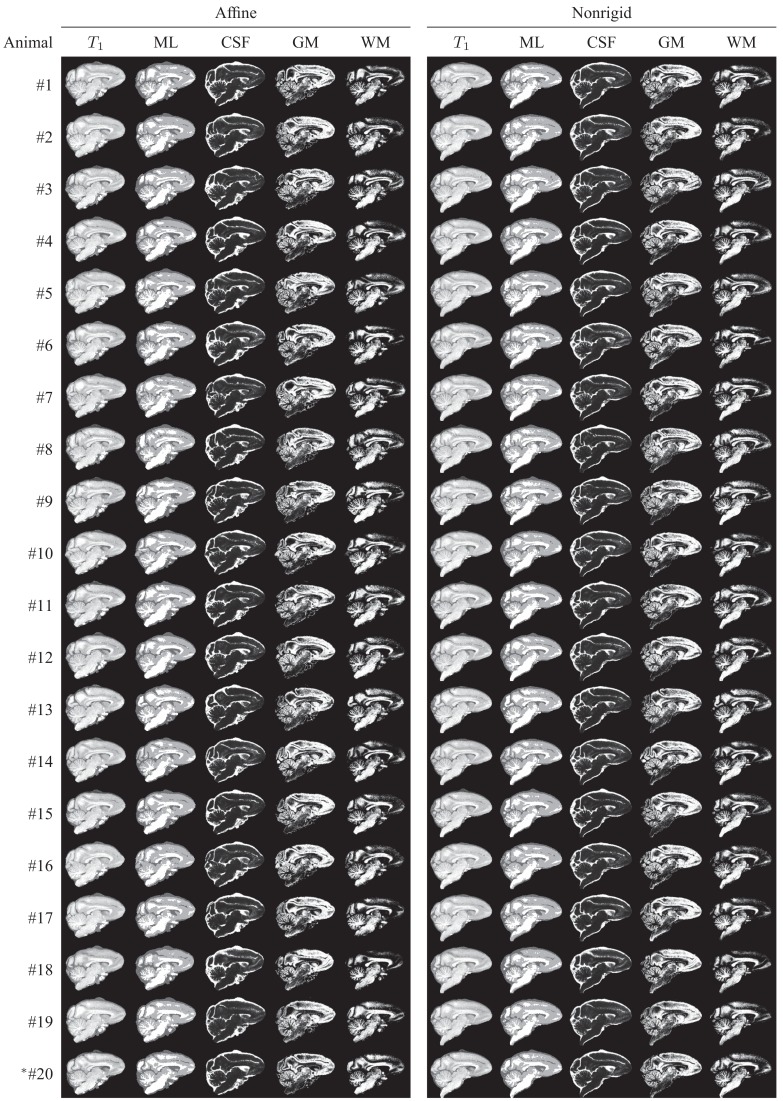
**Animal T1-weighted images, maximum-likelihood segmentations, and tissue probability maps, all reformatted to the INIA19 atlas coordinate system via affine (left column) and non-rigid (right column) registration**. *Animal #20 was the one that was excluded from atlas generation due to abnormal ventricular anatomy.

After computing a coordinate transformation, again via image registration, from the atlas template image to an individual animal image, data from the individual can be mapped into atlas space. This process is commonly referred to as “spatial normalization” and serves the purpose of enabling the pixel- or region-wise comparison of properties across a population. This operation is central to the widely used group analysis technique Voxel-based Morphometry (VBM; Ashburner and Friston, 2000). Figure 10 shows, for illustrative purposes, that the INIA19 atlas is well-suited for spatial normalization using both affine and non-rigid registration methods.

## 5 Conclusion

We have created a new MRI-based template for imaging-based studies of rhesus macaque brains from high-resolution, *T*_1_-weighted structural scans of 19 outbred young to middle-aged adult male animals. The template comprises both whole-head and brain-only unbiased, minimum-deformation template images, tissue probability maps (CSF, gray, and white matter), and a maximum-likelihood tissue label map.

We have initially focused on *T*_1_-based MR contrast on this sex/age group to meet our specific needs for the analysis of brain anatomical changes associated with chronic ethanol exposure. However, the age range chosen for this work (approximately equivalent to 25–42 human years of age) is of broad relevance to other potential work, as dramatic anatomical changes associated with brain maturation are mostly complete, yet it is prior to the appearance of normal and pathology-derived age-related brain changes. Moreover, the approach described here can be readily adapted to incorporate additional imaging modalities, such as *T*_2_-based MR contrast, diffusion-based MR contrast, and PET imaging results, as has been done with the MNI152 (also known as ICBM152) human brain atlas, to which diffusion-weighted information from different subjects than the original 152 was later added (Oishi et al., 2008).

In addition to future incorporation of supplemental image data sets, it is also anticipated that improvements will be made to the NeuroMaps in the future, and other, complementary label maps may also become available. In recognition of such likely developments, the strategy described here was deliberately made in a manner that can accommodate new label maps; and updated INIA19 packages can be distributed, clearly marked by an increasing version number.

The INIA19 template, together with the comprehensive NeuroMaps atlas label map, is made freely available to the scientific community under a Creative Commons attribution license (CC-BY-3.0^9^). All files and documentation are available from the INIA19 home page at http://nitrc.org/projects/inia19/.

## Conflict of Interest Statement

The authors declare that the research was conducted in the absence of any commercial or financial relationships that could be construed as a potential conflict of interest.

## Supplementary Material

The Supplementary Material for this article can be found online at http://www.frontiersin.org/Neuroinformatics/10.3389/fninf.2012.00027/abstract

## Footnotes

^1^http://www.scripps.edu/california/research/inia/index.html

^2^One animal was excluded from atlas construction after image inspection due to abnormally shaped lateral ventricles; see Section “2.2” below.

^3^http://www.fil.ion.ucl.ac.uk/spm/

^4^http://braininfo.rprc.washington.edu/TemplateNeuroMaps.aspx

^5^If, in the future, the NeuroMaps label map receives a significant update or correction, we will propagate the new label map to the INIA19 template using the existing mapping procedure. An updated INIA19 package will then be distributed, clearly marked by an increasing version number.

^6^http://nifti.nimh.nih.gov/nifti-1/

^7^The fastest-varying index of the pixel array corresponds to the left-to-right anatomical direction, the second-fastest to the posterior-to-anterior direction, and the third-fastest (i.e., slice) index to the inferior-to-superior direction.

^8^http://slicer.org

^9^https://creativecommons.org/licenses/by/3.0/
